# Expression Levels of RAD51 Inversely Correlate with Survival of Glioblastoma Patients

**DOI:** 10.3390/cancers13215358

**Published:** 2021-10-26

**Authors:** Christopher Morrison, Eric Weterings, Daruka Mahadevan, Abhay Sanan, Martin Weinand, Baldassarre Stea

**Affiliations:** 1 Department of Radiation Oncology, University of Arizona, Tucson, AZ 85719, USA; chris.morrison@arizona.edu (C.M.); weterings.eric@gmail.com (E.W.); 2 Mays Cancer Center, University of Texas Health, San Antonio, TX 78229, USA; mahadevand@uthscsa.edu; 3 Center for Neurosciences, Tucson, AZ 85718, USA; asanan@neurotucson.com; 4 Department of Neurosurgery, University of Arizona College of Medicine, Tucson, AZ 85724, USA; mweinand@arizona.edu

**Keywords:** RAD51, GBM, NanoString nCounter, gene expression, prognostic marker

## Abstract

**Simple Summary:**

Identifying prognostic and predictive biomarkers for glioblastoma (GBM), a primary brain tumor, is essential in improving patient survival. We utilized gene expression profiling to investigate a uniform population of GBM patients who had been treated with surgery and adjuvant radiation therapy versus normal brain tissue, and identified high RAD51 expression as a poor prognostic marker that is amenable to therapeutic intervention. This observation was confirmed utilizing a publicly available gene expression dataset in a cohort of GBM patients.

**Abstract:**

Treatment failures of glioblastoma (GBM) occur within high-dose radiation fields. We hypothesized that this is due to increased capacity for DNA damage repair in GBM. We identified 24 adult GBM patients treated with maximal safe resection followed by radiation with concurrent and adjuvant temozolomide. The mRNA from patients was quantified using NanoString Technologies’ nCounter platform and compared with 12 non-neoplastic temporal lobe tissue samples as a control. Differential expression analysis identified seven DNA repair genes significantly upregulated in GBM tissues relative to controls (>4-fold difference, adjusted *p* values < 0.001). Among these seven genes, Cox proportional hazards models identified RAD51 to be associated with an increased risk of death (HR = 3.49; *p* = 0.03). Kaplan–Meier (KM) analysis showed that patients with high RAD51 expression had significantly shorter OS compared to low levels (median OS of 10.6 mo. vs 20.1 mo.; log-rank *p* = 0.03). Our findings were validated in a larger external dataset of 162 patients using publicly available gene expression data quantified by the same NanoString technology (median OS of 13.8 mo. vs. 17.4 mo; log-rank *p* = 0.006). Within this uniformly treated GBM population, RAD51, in the homologous recombination pathway, was overexpressed (vs. normal brain) and inversely correlated with OS. High RAD51 expression may be a prognostic biomarker and a therapeutic target in GBM.

## 1. Introduction

Glioblastoma (GBM), a WHO grade IV astrocytoma, is the most commonly diagnosed primary brain tumor in adults [1]. Despite intense research, progress in the treatment of this disease has been modest over the past three decades. The current standard of care, which consists of maximal safe resection followed by 60 Gy of fractionated radiation with concurrent and adjuvant temozolomide (TMZ) for 6 months, was established more than 15 years ago by the Stupp’s NCIC/EORTC trial [2]. Unfortunately, this treatment approach is rarely curative, though it does extend the median overall survival (OS) of patients with glioblastoma by about 2 months, to a total of approximately 14 months [2]. More recently, the use of tumor-treating electrical fields (TTF, Optune, Novocure) has been shown to add about another 5 months to this expected median OS [3], but this treatment modality has a number of barriers to widespread use [4]. Thus, new treatment approaches are needed in order to change the prognosis for this mostly incurable disease.

Although GBM appears homogeneous by histopathology, several molecular alterations have been discovered over the past two decades that result in differing phenotypes. For example, isocitrate dehydrogenase (IDH) gene mutations are an early event that leads to a multitude of downstream epigenetic changes. Patients with IDH mutations have a more favorable prognosis, but this mutation only occurs in about 5% of GBM patients [5]. In addition to somatic mutations, there are also epigenetic changes with clinical significance in glioblastoma. The most well-studied is methylation of the promoter for the O6-methylguanine-DNA methyltransferase (MGMT) gene, which is present in nearly half of GBM patients. This downregulation of MGMT gene expression is both prognostic and predictive of response to temozolomide [6].

The poor clinical outcome of patients with GBM is undoubtedly multifactorial; although these tumors rarely spread through the CSF or outside of the brain, one of the most perplexing features is the glioblastoma’s resistance to standard oncologic treatments, as the majority of tumors treated with radiation and chemotherapy fail within the high-dose radiation fields, despite adequate surgery and maximally tolerated radiation [7]. The underlying mechanism for radiation resistance is also likely multifactorial, involving at least each of the four basic radiobiological principles: fast repopulation of cancer cells [8], presence of hypoxia [9,10], reassortment of cells in the form of cellular quiescence [11], and likely a greater ability for DNA damage repair [12,13]. We focused our investigation on this last principle, and hypothesized that patients with poor response to standard chemo-radiation treatments, resulting in earlier deaths, might be endowed with a greater DNA damage repair capacity compared to those patients that lived longer after receiving identical treatments of surgery, maximal radiation and chemotherapy. To test this hypothesis, we conducted a retrospective investigation of the expression levels of genes known to be important in DNA damage repair in a cohort of GBM patients treated at our institution in a uniform fashion. We first compared the differential expression of DNA repair genes in GBM samples relative to non-neoplastic normal brain tissue controls obtained from temporal lobes of epileptic patients, and then correlated the mRNA expression profiles to the OS of the GBM patients to identify which of the dysregulated DNA damage repair genes were the most strongly associated with survival. Here, we report the findings of our investigation showing that increased expression of DNA repair genes led to increased treatment resistance and decreased survival after standard treatments. More specifically, we report the association of a specific DNA repair enzyme (Rad51) overexpression with decreased survival in both our patient population and in a larger dataset used for validation of our initial observation.

## 2. Materials and Methods

### 2.1. Clinical Study Design, Patient Selection, and Clinical Data Collection

This retrospective study was deemed to meet the criteria for exemption by our Institutional Review Board’s Office for Research and Discovery, Human Subjects Protection Program, under rule 45 CFR 46.101(b), and this decision was filed under protocol #1709802216. We identified 24 patients with a diagnosis of GBM who also had formalin-fixed, paraffin-embedded (FFPE) tissue archived in our institution’s biobank and who had undergone the same treatment of maximal safe resection followed by conventionally fractionated adjuvant radiation (60 Gy) with concurrent and adjuvant TMZ chemotherapy. Deidentified information on patient demographics, treatment and tumor characteristics, and outcomes were retrospectively collected after review of their medical records. IDH1 mutation and MGMT methylation status was assayed at the time of diagnosis, or by the CLIA-certified reference lab at the Mayo Clinic (Rochester, MN, USA) for those patients who had not undergone an analysis at the time of diagnosis. OS was defined as the time between the date of the surgery establishing the diagnosis of glioblastoma and the date of death. Those patients still alive at the time of data collection were censored as of the date of their last physician encounter or their last imaging procedure, whichever came later. Tumor size was defined as the product of the maximal axial anterior-posterior dimension by the maximal orthogonal lateral dimension on preoperative contrast-enhanced T1 MRI scan. The extent of resection was defined as either gross total resection (GTR) or subtotal resection (STR), based on assessment of postoperative MRI by board-certified neuroradiologists. Two KPS groups were defined as either ≥70 or <70.

### 2.2. NanoString Gene Expression Data Collection and Analysis

Formalin-fixed, paraffin-embedded (FFPE) GBM tissue was unarchived and processed by our institutional Tissue Acquisition and Cellular/Molecular Analysis Shared Resource (TACMASR). Given the retrospective nature of this study, which used archived FFPE tissue samples, the method used to quantify gene expression was deemed most critical, as the FFPE process is known to degrade RNA, thus hampering accurate quantification [14,15]. To this end, we selected the nCounter Analysis System from NanoString Technologies (Seattle, WA, USA), which has been shown to effectively and reliably quantify mRNA expression from FFPE tissues at least as well as, if not better than, other techniques such as RT-PCR, microarrays, and RNA sequencing [16,17,18]. RNA was isolated using a Roche HighPure FFPET RNA Isolation spin-column kit from deparaffinized FFPE GBM tissue. Frozen non-neoplastic temporal lobe tissue (archived in RNA-later storage reagent) was used as control; tissue samples were lysed and homogenized, and RNA was isolated by organic extraction and then purified using a Qiagen RNeasy spin-column kit. Purified RNA (300 ng) from FFPE tissues, or 100 ng from frozen non-neoplastic tissue, was hybridized with the gene expression code set probes of an nCounter PanCancer Human Pathways panel (NanoString Technologies, Seattle, WA, USA). Isolation and binding of hybridized probes to an optical cartridge was performed on an automated NanoString nCounter Prep Station. The cartridge was then scanned by means of the nCounter Digital Analyzer to obtain gene-specific probe counts. Raw data was analyzed by means of NanoString Technologies’ nSolver Analysis (version 4.0, Seattle, WA, USA) software, using the Advanced Analytics package (version 2.0, Seattle, WA, USA). Data was normalized against internal positive and negative controls, as well as 40 housekeeping genes, using the geNorm algorithm [19] per the manufacturer’s instructions [20]. For control, we used non-neoplastic tissue from archived cortical brain tissue, obtained and frozen as previously described [21], from 12 patients who had undergone lateral temporal lobe resections as part of a treatment for epilepsy. No clinical information was obtained from this cohort of patients. The data discussed in this publication have been deposited in NCBI’s Gene Expression Omnibus and are accessible through GEO Series accession number GSE186057 (https://www.ncbi.nlm.nih.gov/geo/query/acc.cgi?acc=GSE186057, accessed on 26 November 2019)

### 2.3. Statistical Methods

Differential expression (DE) of individual genes, with adjusted *p*-values to account for multiple comparisons with the Benjamini–Yekutieli method [22], was done by the provided nSolver software. Cox proportional hazards (CPH) models and Kaplan–Meier (KM) survival estimates were also generated in the open-source software R (version 3.3.1) and R Studio (version 1.0.136) [23]. For the sake of the CPH models, one patient with an indeterminate IDH-1 mutation status was categorized as positive, while patients with indeterminate MGMT methylation status were categorized as negative, because their measured MGMT expression levels were more consistent with unmethylated. Comparison of normalized and log2-transformed RAD51 expression levels between MGMT methylated and un-methylated patients was conducted with a Student’s *t*-test.

### 2.4. Validation in an Independent Dataset

We obtained gene expression data, quantified by the same NanoString nCounter platform, with clinical annotations, from the post hoc analysis of the AVAglio trial by Sandmann et al. [24] by using the NCBI Gene Expression Omnibus (GEO) [25,26] (https://www.ncbi.nlm.nih.gov/geo/query/acc.cgi?acc=GSE84010, accessed on 26 November 2019). The AVAglio trial was a randomized placebo-controlled trial that investigated the addition of bevacizumab in the adjuvant setting to the current standard of care of radiation and temozolomide. To match this patient population to the one used in our own dataset as much as possible, we only included the patients on the placebo arm of the AVAglio dataset (no bevacizumab) and, as we had done for our cohort, excluded patients who had their primary tumor biopsied rather than resected. This left 162 patients to be analyzed. The raw expression data were normalized using the nSolver Advanced Analytics software exactly as done with our own institutional dataset.

## 3. Results

### 3.1. Patients, Tumor, and Treatment Characteristics

The median age of the 24 GBM patients in our dataset was 63.5 years, and the median OS of the entire cohort was 12.3 months. Other demographic factors and tumor characteristics known to be prognostically relevant in GBM are shown in Table 1. One of the 24 patients (4.2%) had an IDH1 mutation, while another sample was indeterminate on immunohistochemistry. Six patients (25%) had their MGMT promotor methylated, while two were deemed indeterminate.

### 3.2. Differential Expression of Pathways and Individual Genes Involved with DNA Damage Repair

**Figure 1:**
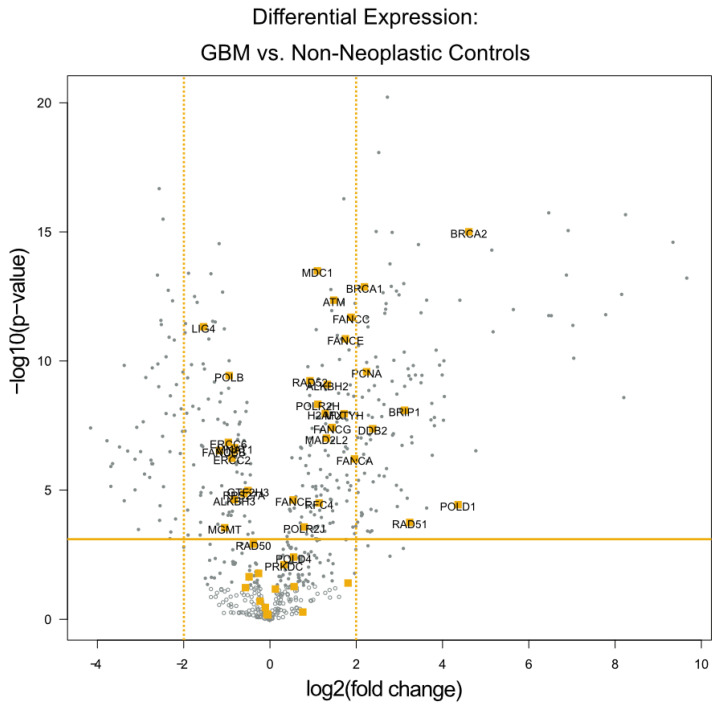
Differential Expression of Individual DNA Damage Repair Genes. A volcano plot of differentially expressed genes with the genes categorized by the NanoString software as being related to DNA damage repair highlighted in orange. The x-axis displays the fold change in DE laterally, while the y-axis displays decreasing *p* values vertically. The orange horizontal line depicts the *p*-value threshold of 0.01, and the orange vertical lines depict the 4-fold change threshold used to identify the most dysregulated DNA damage repair genes.

The differential expression analysis of the specimens from these 24 patients identified seven genes with expression levels at least 4-fold higher relative to the 12 temporal lobe control tissue samples (all *p* values < 0.01). All of these seven genes are known to be involved in DNA damage repair. These genes are highlighted in orange within the volcano plot (Figure 1), and listed in Table 2. Notably, four of the seven genes (BRCA1, BRCA2, BRIP1, and RAD51) play a significant role in the homologous recombination process, while the other three serve more global roles in DNA damage repair.

### 3.3. Survival Analyses

**Figure 2:**
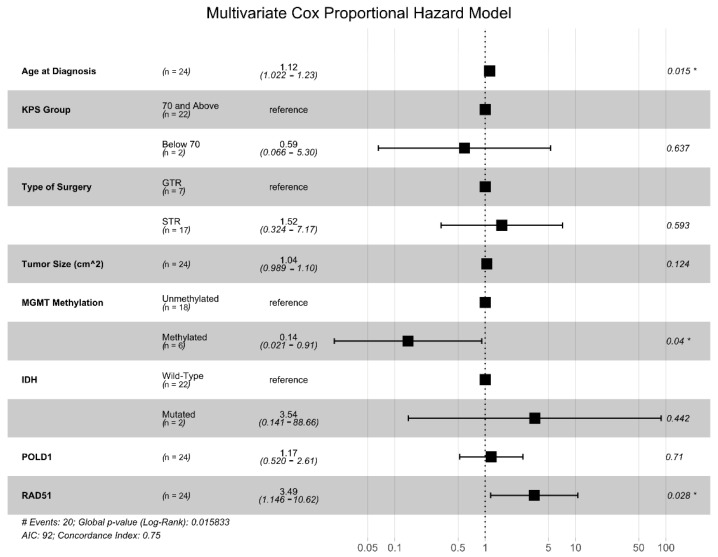
Multivariate Cox proportional hazards (CPH) model for OS. Multivariate CPH model including known clinical and genetic prognostic factors, as well as the seven DNA damage repair genes with at least a 4-fold differential expression and an adjusted *p* < 0.01 in the GBM vs. non-neoplastic comparison. * denotes variables with *p* values < 0.05.

We generated univariate CPH models for each of the seven DNA damage repair genes identified above, and only two were found to be significantly associated with OS: POLD1 (HR = 2.01, *p* = 0.046) and RAD51 (HR = 2.26, *p* = 0.017). Next, we generated a multivariate Cox proportional hazards (CPH) model that included the expression levels of POLD1 and RAD51 and other common clinical prognostic factors (patient age, KPS group, tumor size, extent of resection, IDH mutational status, and MGMT promotor methylation status) (Figure 2). This analysis showed that RAD51 expression level was independently associated with an increased risk of death (HR = 3.49, *p* = 0.028) along with increasing age (HR = 1.12, *p* = 0.039), while MGMT promotor methylation conferred a survival advantage (HR = 0.14, *p* = 0.04).

**Figure 3:**
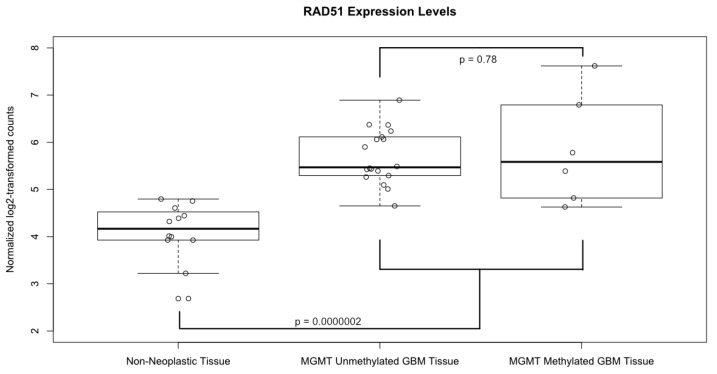
Expression levels of RAD51. Boxplot comparing the normalized, log2 transformed, RAD51 expression levels between GBM tissue samples, both MGMT methylated and unmethylated, and non-neoplastic controls. Solid horizontal lines represent the median value of each group; boxes show interquartile range (25–75th percentiles), while whiskers show the range of values contained within 1.5 times the interquartile range. Values from each individual sample are superimposed as hollow circles.

A boxplot of the normalized and log2 transformed expression levels of RAD51 from all of the 24 samples is shown in Figure 3. The average log2-transformed expression level of RAD51 in GBM was significantly higher than in the non-neoplastic control tissues (5.73 vs. 4.09, *p*=0.0000002). Of note, there was no significant difference in RAD51 levels in GBM samples based on MGMT methylation status (*p* = 0.78). In fact, the sample with the lowest RAD51 expression level and the highest level came from patients with methylated MGMT promotors. Likewise, the only one IDH1 mutant tumor in our dataset had low RAD51 levels, while the patient with equivocal IDH1 staining had relatively high RAD51 levels.

**Figure 4:**
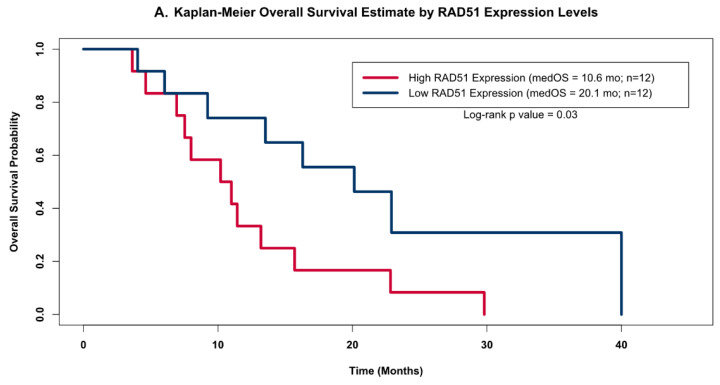

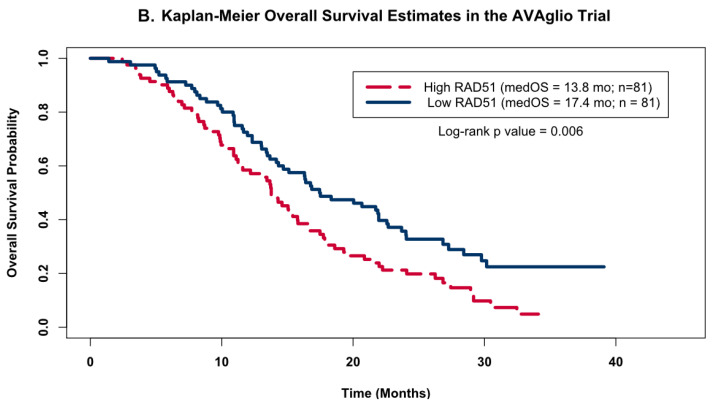
Kaplan–Meier OS Models. Kaplan–Meier OS probability estimates comparing GBM patients grouped by RAD51 expression levels. High expression (red) levels were defined as values above the cohort’s median log2-transformed expression level, while low (blue) was defined as below the median expression. (**A**) shows the KM curves for the patients from this study (high Rad 51 = 12, low Rad51 = 12); (**B**) shows external validation using the same analysis for the publicly available independent dataset obtained as part of the AVAglio trial (high Rad 51 = 81, low Rad51 = 81).

The distribution of RAD51 levels across the GBM samples suggested that there may be two distinct clusters of RAD51 expression levels within this small but homogenously treated population of GBM patients (open circles in Figure 3); therefore, we grouped patients into high and low RAD51 expression groups based on whether or not each sample’s expression level was above or below the cohorts’ median value. When we compared the OS estimates of these two groups using the Kapan–Meier (KM) method (Figure 4A), we found that the group of patients with high RAD51 expression had significantly shorter OS compared to the group with low RAD51 expression levels (10.6 months versus 20.1 months, respectively; log-rank *p*-value = 0.03).

### 3.4. External Validation

To validate the observed association between RAD51 expression levels and OS, we investigated this relationship in a publicly available dataset from the AVAglio trial, a dataset that also had its gene expression quantified by the NanoString nCounter Analysis system. As was done with our cohort of 24 patients, we divided the patients from the AVAglio trial into high or low RAD51 groups based on whether their individual expression levels were above or below the cohort’s median expression level, and then compared their KM OS estimates. Analysis of the patients on the control arm of the AVAglio trial (shown in Figure 4B) confirmed our initial observation that high levels of RAD51 were associated with worse OS; patients with high RAD51 levels had a median OS of 13.8 months, while patients with low levels of RAD51 had a median OS of 17.4 months (log-rank *p* value = 0.006).

## 4. Discussion

In this clinical–pathological study of the expression profiles of genes related to DNA damage repair in a patient population with glioblastoma, we identified several genes from the homologous recombination (HR) pathway to be significantly overexpressed in glioblastoma tissue samples relative to non-neoplastic brain tissue. To our knowledge, this is the first study to demonstrate an increase in DNA repair gene expression over “normal” brain tissues. This finding could explain why these tumors are so resistant to radiotherapy. Homologous recombination is indeed one of two main pathways used to repair double-stranded DNA breaks caused by radiation [27]. The other significant finding reported here is that the expression level of a specific gene within the HR pathway, RAD51, appeared to be inversely correlated with the overall survival of GBM patients. The greater the expression, the worse the survival, consistent with our hypothesis that the apparent radiation resistance of GBM was partially due to a greater ability to repair DNA double-strand breaks induced by radiation. While increased RAD51 expression has been previously reported in GBM cell lines, to our knowledge, this is the first report to correlate its expression with survival of patients with GBM. It is noteworthy to mention here that RAD51 plays a crucial role within the HR process, as it facilitates DNA strand exchange between the broken strands of DNA and the unbroken template strand in order to allow DNA polymerases to replicate the homologous sequence to repair the double-strand break [28]. A possible mechanistic explanation for this upregulation of RAD51 in GBM cell lines comes from a recent published work on CHD4, an ATPase and member of the nucleosome remodeling and deacetylase (NuRD) complex, which also regulates the expression of RAD51; not only the CHD4 complex goes to sites of DNA damage, but it appears that CHD4 also upregulates expression of RAD51 [29].

The upregulation of RAD51 reported in this study is also consistent with previously published in vitro studies showing increased expression of RAD51 mRNA [30] and protein levels in glioma cell lines [31] compared with normal human astrocytes in culture. In addition, overexpression of RAD51 protein has been reported using immunohistochemistry (IHC) in GBM-derived FFPE tissue samples [32] and other solid malignancies [33]. Other investigators have attempted to correlate RAD51 protein levels and survival of GBM patients using IHC-based methods, and found conflicting results [32,34]. To our knowledge, this study is the first to correlate RAD51 mRNA expression levels from patient-derived tissues to the survival of those patients using a modern, objective, and quantitative methodology with a normal tissue control. Our findings demonstrated increased RAD51 levels correlated with worse patient outcomes clinically, and confirmed our hypothesis that increased repair capacity of DNA damage could be a mechanism of treatment resistance in GBM and possibly other cancers. The survival difference based on RAD51 expression levels noted in our small set of patients was confirmed in a much larger cohort of GBM patients treated on a national protocol. Additional evidence that RAD51 plays an important role in the radiation resistance of this cancer comes from several pre-clinical in vitro studies showing that inhibition of RAD51 increases radiation sensitivity of GBM cell lines [30,31,34,35].

The limitation of the study reported here was in its retrospective nature and small sample size. However, despite these limitations, the small but biologically homogenous sample size of 24 GBM patients provided more than sufficient power to detect differentially expressed genes between GBM and non-neoplastic brain tissue, although it may have limited the ability of the Cox proportional hazards models from detecting real associations between GBM patient OS and the expression levels of other DNA repair genes. It was also less than ideal that the control tissues came from fresh frozen samples while the GBM samples were from FFPE tissue, but the NanoString platform has already been shown to have a high correlation between the frozen and the FFPE sample types [17,18], therefore the difference in counts due to the difference in sample types should be minimal.

In conclusion, our findings, combined with the work of others, suggest that RAD51 expression levels could be a clinically informative prognostic biomarker for GBM patients; this finding may offer additional prognostic value in addition to other established prognostic indicators (IDH mutation and MGMT promoter methylation). Furthermore, the findings reported here also have therapeutic implications; if the prognostic role of RAD51 is confirmed by a prospective study, adaptive management strategies could be designed based on the level of RAD51 expression, such as clinical trials of different radiation regimens and/or intensity; for example, patients with high repair capacity could be assigned to receive hypo-fractionated radiation therapy to compensate for the increased cellular capacity to repair DNA double-strand breaks [36,37]. Indeed, hypo-fractionated radiation could theoretically be more effective in patients with increased DNA damage repair mechanisms, a hypothesis currently under investigation [38,39]. Finally, and more importantly, the results reported here point the way toward a potential therapeutic target that could be exploited to increase the sensitivity of GBM to radiation, a concept that already has support in in vitro studies of inhibition of the c-MET receptor tyrosine kinase, which in turn leads to a decrease in Rad51 expression levels, thereby increasing the radio-sensitivity of GBM cell lines [35]. Similarly, another tyrosine kinase inhibitor, amuvatinib (also known as MP470), has also been shown to downregulate RAD51 expression in vitro, and acts synergistically with radiation in GBM cell lines [40].

## 5. Conclusions

Utilizing a commercially available mRNA-based gene expression profiling, this study identified RAD51, a gene involved in the homologous recombinant pathway of DNA repair, as being significantly overexpressed in GBM-derived tissues relative to non-neoplastic brain tissue. The expression level of this repair gene was also inversely correlated with the overall survival of GBM patients. Therefore, measurement of RAD51 mRNA expression from surgically removed specimens could be used as a molecular biomarker to help refine the prognosis of patients with GBM. Finally, inhibition of the RAD51 protein may provide a mechanism to overcome radiation treatment resistance in GBM and other cancers overexpressing RAD51.

## Figures and Tables

**Table 1:** Patient, tumor, and treatment characteristics.

Demographics	GBM Patients (*n* = 24)
Males/Females	18/6
Median Age at Dx (Range)	63.5 (41–80)
Median KPS (Range)	80 (50–90)
Avg Tumor Size in cm^2^ (Range)	21.2 (1.9–41.2)
Median OS (Months)	12.3
Extent of Surgery (% of pts)	
GTR	29.2
STR	70.8
IDH1 Mutation Status	
Wildtype	22
Mutant	1
Indeterminate	1
MGMT Promotor Methylation Status	
Unmethylated	18
Methylated	6
Indeterminate	2
Completion of concurrent TMZ (% of pts)	95.8
Range of RT Dose (Gy)	60–75

**Table 2:** DNA repair genes with significant differential expression.

Gene	log2(DE)	*p* Value	Function
BRCA2	4.61	9.9 × 10^−16^	Homologous Recombination
POLD1	4.36	3.7 × 10^−5^	DNA Polymerase Delta
RAD51	3.24	1.8 × 10^−4^	Homologous Recombination
BRIP1	3.12	8.1 × 10^−9^	Homologous Recombination
DDB2	2.38	4.2 × 10^−8^	DNA damage-binding protein 2
PCNA	2.24	2.6 × 10^−10^	Co-factor for POLD1
BRCA1	2.19	1.3 × 10^−13^	Homologous Recombination

## Data Availability

The datasets generated and/or analyzed during the current study are available from the corresponding author upon request.
